# The effects of apoptosis vulnerability markers on the myocardium in depression after myocardial infarction

**DOI:** 10.1186/1741-7015-11-32

**Published:** 2013-02-08

**Authors:** Yiming Wang, Xingde Liu, Dongfeng Zhang, Jianhui Chen, Shuzheng Liu, Michael Berk

**Affiliations:** 1 Department of Psychiatry, Hospital Affiliated to Guiyang Medical University, 28 Guiyi Street, Guiyang City, 550004, Guizhou, China; 2 School of Medicine, Deakin University, 1 Gheringhap Street, Geelong, VIC 3220, Australia; 3 Department of Cardiology, Hospital Affiliated to Guiyang Medical University, 28 Guiyi Street, Guiyang City, 550004, Guizhou, China; 4 Department of Neurology, The General Hospital of Hebi Coal Corp, 84 Honggi Street, Hebi City, 458000, Henan, China; 5 Department of Psychiatry, The University of Melbourne, Level 1, North Block Main, Building Royal Melbourne Hospital, Parkville, VIC 3050, Australia; 6 Orygen Youth Health Research Centre, Centre for Youth Mental Health, 35 Poplar, Road, Parkville VIC 3052, Australia; 7 Florey Institute of Neuroscience and Mental Health, Level 3, Alan Gilbert Building, 161 Barry Street, University of Melbourne, Parkville, VIC 3010, Australia

**Keywords:** major depressive disorder, myocardial infarction, apoptosis, myocardium, stress, cardiac, comorbidity.

## Abstract

**Background:**

There is an increased incidence of major depressive disorder (MDD) in individuals after myocardial infarction (MI), but the pathophysiological processes mediating this association are unclear. Our previous study demonstrated an increase in pro-apoptotic pathways in the myocardium and hippocampus in MDD, which was reversed by venlafaxine. This study aimed to attempt to confirm the effects of apoptosis vulnerability markers on the myocardium in a model of depression after myocardial infarction.

**Methods:**

Rats were divided into four groups: sham (N = 8), depression (N = 8, chronic mild unpredictable stress and separation were used in the depression group), MI (N = 13) and post-MI depression (N = 7). The rats in all four groups underwent the same open field and sucrose preference behavioral tests. Evan Blue staining was used to determine the area at risk of myocardial infarction in the left ventricle, and 2,3,5-triphenyl tetrazolium chloride (1.5% TTC) dye was used to detect the size of the myocardial infarction. The expression of bax and bcl-2 protein in the myocardium was investigated by immunohistochemistry, and the mRNA expression of bax, bcl-2 and caspase-3 in the myocardium was investigated by real time RT-PCR. Apoptosis was estimated in the myocardium by measuring the Bax:Bcl-2 ratio.

**Results:**

In the depression and post-MI depression rats, there were significantly decreased movements and total sucrose consumption, modeling behavioral deficits and an anhedonic-like state. In terms of myocardial infarction size, no difference was seen between the MI and post-MI depression groups. There was an up-regulated Bax:Bcl-2 ratio in the depression, MI and post-MI depression groups. Furthermore, in the latter group, there was a greater up-regulated Bax:Bcl-2 ratio. However, caspase-3 did not differ among the four groups.

**Conclusions:**

These results of this animal model suggest that active pro-apoptotic pathways may be involved in the nexus between myocardial infarction and depression. This mechanism may be germane to understanding this relationship in humans.

## Background

Depression is a major contribution to the global disease burden, and is widely accepted as an independent risk factor in patients with coronary artery disease (CAD). Comorbidity is associated with poorer outcomes [1-5]. Myocardial infarction (MI) is the most serious clinical form of CAD [6]. There is an increased incidence of major depressive disorder (MDD) (15 to 30%) after myocardial infarction (MI) [7]. Equally, depression is linked to a 2.0- to 2.5-fold increased risk in new cardiovascular events and increased cardiac mortality [8-10]. Patients frequently develop depressive symptoms after an acute MI, and depression is linked to an increased long-term risk of morbidity and mortality [11]; however, the pathophysiological mechanisms underpinning the relationship between MI and depression remain poorly understood.

Apoptosis or programmed cell death is a process of ordered, active, non- inflammatory cell death. Bcl-2 is one member of a family of genes which can be divided into two categories according to their effects on apoptosis, one group promoting apoptosis, including Bax, Bak, Bad and Bcl-xS, a second group inhibiting cell death pathways, including Bcl-2 and Bcl-xl [12,13]. Of these proteins, Bcl-2, Bax and Bcl-x are the best characterized genes in the Bcl-2 family [14]. In the presence of stress, Bcl-2 family proteins congregate at the outer mitochondrial membrane and play a role in the regulation of apoptosis. Pro-apoptotic Bax and Bak undergo conformational changes and Bax translocates from cytosol to mitochondria via homo-oligomerization with cell stress signals [15].

The Bax/Bcl-2 ratio is a measure of a cell's vulnerability to apoptosis, a higher Bax/Bcl-2 ratio is associated with a greater vulnerability to apoptotic activation, and up-regulation of the Bax/Bcl-2 ratio suggests greater apoptotic activity [16,17]. Antidepressants, such as sertraline, can modulate depression-induced behavior and biochemical markers after myocardial infarction [18].

Caspases are a family of inactive proenzymes. Generally, there are two pathways in caspase family proteases which can be activated: one is the death receptor-mediated pathway that is death signal-induced; another is the mitochondrion-mediated stress-induced pathway that truncates a pro-apoptotic Bcl-2 family member [19].

Caspase-3 is a protein that regulates apoptosis by inducing the cleavage of the key cellular proteins and alters cell integrity. The role of caspase 3 in apoptosis is to activate the stages of cellular death in a non-traumatic manner. Activation of caspase-3 is another pathway to apoptosis.

It has been recently demonstrated that the release of pro-inflammatory cytokines in rats can induce limbic system apoptosis after an acute myocardial infarction [20-22]. Wann [23] has shown behavioral changes and an increased Bax/Bcl-2 protein ratio in limbic areas in rats with post-MI depression, suggesting a role of apoptotic events, which is similar to human findings.

Our previous study [24] demonstrated that in rats with chronic mild stress (CMS), there were significant behavioral deficits, an increase in Bax levels and a decrease in Bcl-xl levels in the myocardium and hippocampus, suggesting an increase in pro-apoptotic pathways. This was reversed by venlafaxine, which is an antidepressant of the serotonin-norepinephrine reuptake inhibitor (SNRI) class; however, in models of post-MI with depression, the role of apoptosis vulnerability markers in the myocardium is unclear, in particular associations with apoptotic pathways. We consequently hypothesized that active pro-apoptotic pathways in the myocardium may be involved in the nexus between cardiovascular disorders and depression.

To investigate this issue, we developed a valid model of myocardial infarction, alone and in conjunction with an anhedonic-like state in rats. This study aimed to attempt to evaluate vulnerability markers of myocardial apoptosis, specifically the Bax:Bcl-2 ratio and caspase-3 levels in the myocardium post-MI depression, to clarify the molecular mechanisms and, as well, confirm whether the co-occurrence of myocardial infarction with MDD is associated with greater activation of apoptosis pathways.

## Methods

### Subjects

Male Sprague-Dawley rats (N = 36, weighing 250 g ± 20 g) were used for the experimental procedures. Rats were allowed to adapt to the surroundings for one week prior to the commencement of the experiment. Each rat was housed singly at room temperature (22 to 25°C), humidity (40 to 50%), the light period was 12 h from 8 a.m., and food and water were freely available. Rats were divided into four groups: (i) sham group (N = 8), (ii) depression group (N = 8), (iii) MI group (N = 13), (iv) post-MI depression group (N = 7). The rats in all four groups performed the same open field and sucrose preference behavioral tests. In the depression and post-MI depression rats, there were significantly decreased movements and reduced total sucrose consumption, modeling behavioral deficits and a depressive-like anhedonic state [25]. The model of post-MI depression, in which decreased movement and an anhedonic- state occurred, was observed in 7 out of 20 myocardial infarction rats in our experiment. Animals were managed in accordance with the American Psychological Association (APA) ethical standards in the treatment of rats and National Institute of Health and Guide for the Care and Use of Laboratory Animals (NIH Publications No. 80-23) revised in 1996, and the study was approved by the ethics Committee on the Guidelines for Animal Experiment at Guiyang Medical University. Efforts were made to minimize the number of animals used and their suffering.

### Myocardial infarction model (MI group)

**Figure 1:**
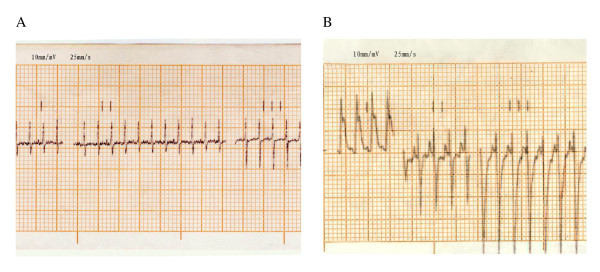
**The electrocardiogram of the rats**. **A**: Electrocardiogram in the normal rat. **B**: ST-segment elevation in leads I in myocardial ischemia rat.

In this study, we used the myocardial infarction model of ischemia described by Wu *et al*., which is a validated animal model of myocardial infarction [26]. Surgeries were performed in the morning. Twenty- two rats were anesthetized by 10% chloral hydrate (0.3 ml/100 g), intubated, and maintained on a ventilator (R: 60 times/minute). A thoracic incision of about 2.0 cm was made, and the base of the heart was exposed by retractors in the central region of the rib cage. The myocardial infarction model was induced by ligating the left anterior descending coronary artery 2 mm from the tip of the left auricle by polypropylene suture for 40 minutes. Rats were monitored by ECG (electrocardiogram) before and after coronary artery ligation. Ischemia was confirmed by ST-T segment elevation in ECG recordings performed on the rats (Figure 1) [27], and after the myocardial surface became blanched, the animal was returned to its home cage after the ligation was removed and the thorax sutured for 14 days. In the sham group, eight rats were operated on using the same protocol, except the coronary artery was not ligated. After the surgery, all rats were administered an analgesic (2 mg/kg butorphanol tartrate, s.c. every 8 h during the 24 h after surgery) and an antibiotic (10,000 IU penicillin G, i.m.). The rats did not show any signs of cardiac failure nor arrhythmias during the myocardial infarction procedure. The percentage of rats surviving the MI induction procedure was 90%.

### Chronic mild stress model (depression)

In this study, the chronic mild stress model was used as a validated animal model of depression [28]. Chronic mild unpredictable stress and separation were used in the depression group for 21 days. Eight rats were given the following stressors [25,29], consisting each week of repeated periods of confinement to a small (38 × 20 ×16 cm) cage, restraint (1 h), water deprivation (24 h), food deprivation (24 h), isolation (24 h), flashing light (3 h), forced cold water swimming (10 minutes) and were group-housed in a soiled cage overnight. Individual stressors and length of time used each day are listed in Table 1. Stressors were used daily in a random and unpredictable order for 21 days.

**Table 1:** Schedule of chronic mild stress

	Monday	Tuesday	Wednesday	Thursday	Friday	Saturday	Sunday
Stressor used	Restraint	Water deprivation	Food deprivation	Isolation	Flashing light	Forced cold water swimming	Group-housed in soiled cage
Duration	1 h	24 h	24 h	24 h	3 h	10 minutes	overnight

### Open field test and sucrose consumption test

**Figure 2:**
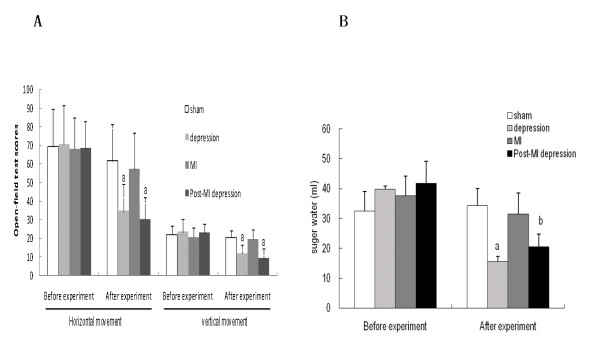
**The behavioral changes in open-field and sucrose consumption test**. Rats in both the depression and post-MI (myocardial infarction) depression groups showed significantly lower scores of both horizontal and vertical movement compared with the sham and MI groups (^a ^*P *< 0.01) (**A**). The total consumptions of sucrose was significantly lower in the depression and post-MI depression groups compared to the sham and MI groups after the experiment (^a ^*P *< 0.01, ^b ^*P *< 0.05) (**B**).

The behavior and anhedonic-like state of all rats were detected by the open field test [30] and sucrose consumption test [31]. The behavioral response to a new environment and activating behavior of rats was detected by the open field test [32], which has been used as an indicator of emotional state. This includes assessment of horizontal movements (the total number of crossing squares) and vertical movements (grooming and rearing) during a five-minute period. The former can be used as a proxy of emotional activity; the latter is regarded as a measure of exploratory activity to novel environments [33]. A special white square (80 × 80 × 40 cm), which has 25 sectors with black stripes on the ground, was used. Animals were separately put in the same central sector, and their activity was recorded during a five-minute period by an installed video camera. Observers analyzed the results of videotapes. Rats in both the depression and post-MI depression groups showed significantly lower scores of both horizontal and vertical movement compared with the sham and MI groups (Figure 2A), suggesting similarly decreased behavioral indices in the post-MI depression and depression groups.

Sucrose consumption is a rat model of an anhedonia-like state [34]. Every cage was offered a bottle of water and a bottle of 1% sucrose water. Total sucrose consumption was measured after 60 minutes. The test was begun 23 h after water and food deprivation, and the experiment was begun, and on day 15 after surgery [23] and the day after the end of the chronic mild unpredictable stress procedure [28]. Before the experiment, there were similar levels of sucrose water consumption between the four groups; however, after the experiment, the total consumptions of sucrose was significantly lower in the depression and post-MI depression groups compared to the sham and MI groups (Figure 2B), suggesting the post-MI depression group was similar to the depression group in terms of developing an anhedonic-like state.

### Myocardial infarct size

The rats were sacrificed by decapitation 24 h after completion of the behavioral tests, prior to which horizontal movements, vertical movements and sucrose consumption tests were recorded according to the experimental protocol. The heart was removed, and the aorta was cannulated and washed with saline, the left anterior descending coronary artery was ligated again at the same site, and the aorta was infused with 2 ml of 0.5% Evan's blue to determine the extent of the non-colored ischemic risk area, then the myocardium was bisected into two parts from the apex to the base along the left anterior descending coronary artery, which was frozen at -80°C for five minutes, and then sliced into 2 mm transverse sections and stained using 2,3,5-triphenyl tetrazolium chloride (1.5%TTC) and myocardial infarction size (×2) was thus confirmed [21]).

### Tissue preparation

The rest of the myocardium was washed using DEPC (diethylpyrocarbonate) H_2_O. The anterior myocardial regional tissue sample (50 mg), which was non-colored tissue using Evan's blue was separated from the edge of the myocardial infarct risk area of the left ventricle. Tissue was frozen using liquid nitrogen and stored at -80°C for future real time RT-PCR use; the remainder was put into 4% paraformaldehyde for 12 h, paraffin embedded, and sectioned (thickness of 4 μm) for immunohistochemical staining.

### The expression of Bax, Bcl-2 and caspase-3 mRNA was detected by real time RT-PCR

Total RNA was extracted from the samples by the TRIZOL reagent (Shanghai ShengGong Biological Engineering Services Co., LTD, Shanghai, China). Primers were synthesized (Shanghai ShengGong Biological Engineering Services Co., LTD, Shanghai, China). cDNA was synthesized (Invitrogen Corp., San Diego, California USA) by combining the components (5× VILO™ Reaction Mix 4 μl, 10× Superscript Enzyme Mix 2 μl, RNA (up to 2.5 μg) Xμl and DEPC-treated water to 20 μl). Tube contents were gently mixed and incubated at 25°C for 10 minutes. Tubes were incubated at 42°C for 60 minutes. The reaction was terminated at 85°C at 5 minutes. cDNA was amplified with Power SYBR Green PCR Master Mix (Roche Molecular Biochemicals, Basel, Switzerland) for each gene, by the Applied Biosystems StepOne™ and Stepone Plus™ real-time PCR System (Foster City, California, USA): pre-denaturation 95°C for 10 minutes, denaturation 95°C for 15 s, annealing 60°C for 1 minute, extending 72°C for 1 minute, denaturation and annealing for a total of 40 cycles, extending again 72°C for 10 minutes. PCR products were analyzed by quantitative high-resolution DNA melting analysis. β-actin was used as an internal standard. Table 2 shows Primer series of Bax, Bcl-2 and β-actin, caspase-3 gene.

**Table 2:** Primer series of Bax, Bcl-2, β-actin and caspase-3

Genes	upstream (5'-3')	downstream (5'-3')	Bp
Bax	GCGATGAACTGGACAACAACAT	TAGCAAAGTAGAAAAGGGCAACC	153
Bcl-2	ATGTGTGTGGAGAGCGTCAACC	CCAGGAGAAATCAAACAGAGGC	174
β-actin	ATGGTGGGTATGGGTCAGAA	ACCCTCATAGATGGGCACAG	375
caspase-3	TGGTTCATCCAGTCGCTTTGT	CAAATTCTGTTGCCACCTTTCG	103

### Quantitative measure of Bcl-2 and Bax protein by immunohistochemical staining

For Bax and Bcl-2 protein determination [35], immunohistochemical staining kits for Bax, Bcl-2 were provided by Wuhan Boster Bioengineering Co., Ltd. (Wuhan, China). The sections (thickness of 4 μm) were de-waxed and rehydrated with freshly distilled water. At room temperature, samples underwent inactivation of endogenous peroxidase for 10 minutes and were washed with distilled water for 2 minutes (three times), then immersed in 0.1 mol/L citrate buffer solution (pH = 6.0) and heated in a microwave oven until boiling (twice, with a 5-minute interval). After refrigerated flushing three times with phosphate buffered saline (PBS) for 2 minutes, antigen-retrieval buffers were added for 10 minutes and tissues were then flushed three times with PBS for 2 minutes. At room temperature, samples were non-specifically blocked with normal goat serum for 20 minutes. After removal of the goat serum, rabbit anti-Bax, Bcl-2 antibody was added (incubated for 60 minutes at 37°C). Biotinylated goat anti-rabbit antibody immunoglobulin G was applied for 20 minutes and flushed with PBS for 2 minutes (three times), then the tissues were placed in a streptavidin-biotin-enzyme complex reagent, incubated for 20 minutes at 37°C, after flushing with PBS for 5 minutes (four times), samples were stained with diaminobenzidine for 25 minutes and washed for 3 minutes. Then samples were stained with hematoxylin, and were dehydrated and mounted for microscopic examination. Images of the sections were obtained (400 X) using the *Image-Pro Plus *4.5 software (Media Cybernetics, Silver. Spring, USA) with brown cytoplasmic staining under light microscopy indicating a positive reaction of Bax, Bcl-2. The myocardial infarction border zone was chosen using a 10 high power fields (400×), in which the mean number of bax and bcl-2 positive cells were obtained. The Bax:Bcl-2 ratio was also confirmed.

### Statistical analysis

The analyses were conducted using IBM SPSS 19.0 analysis software (SPSS Inc, Chicago, Illinois, USA). One-way ANOVA test was performed to examine the data among groups (mean ± SEM) with the Bonferroni *post-hoc *tests for multiple comparisons. There was a significant difference (*P *< 0.05) and all the statistical tests were two-tailed.

## Results

### Infarct size

The myocardial infarct risk area of the left ventricular area was 65 ± 2% (mean ± SEM) indicated by Evan Blue staining. The myocardial infarct size in the myocardial infarct risk area was similar in both groups: (MI group 43.2 ± 1.9%; post-MI depression group 45.6 ± 2.6%). No difference was seen between the MI and post-MI depression groups.

### The expression of Bax and Bcl-2 mRNA and Bax/Bcl-2 ratio

The ratio of Bax:Bcl-2 (mean ± SEM) in the depression, MI and post-MI depression groups was statistically significantly larger than in the sham group (*P *< 0.05). The ratio of Bax-Bcl-2 was significantly larger in MI group than the depression group *(P *< 0.05), and this ratio was significantly greater in the post-MI depression group compared with the MI group (*P *< 0.05) (Table 3).

**Table 3:** The expression of caspase 3, Bax and Bcl-2 mRNA and Bax/Bcl-2 ratio (mean ± SEM, copies/g RNA)

Groups	Casp-3 mRNA	Bax mRNA	BcL-2 mRNA	Bax/bcl-2
Sham (8)	0.66 (0.34)	2.85 (0.58)	0.88 (0.19)	3.29 (0.67)
Depression (8)	0.67 (0.08)	10.73 (1.20)^a^	1.97 (0.26) ^a^	5.55 (1.28) ^b^
MI (13)	0.92 (0.55)	23.23 (1.97)^a, c^	3.12 (0.10) ^a, c^	7.47 (0.86) ^a, d^
Post-MI Depression (7)	0.95 (0.42)	46.79 (2.75)^a, c, e^	5.08 (0.55) ^a, c, e^	9.26 (0.67) ^a, c, f^
***F***	0.481	333.978	93.541	24.171
***P***	0.704	0.000	0.000	0.000

The ratio of Bax:Bcl-2 (mean ± SEM) in the depression, MI (myocardial infarction) and post-MI depression groups was significantly larger than in the sham group (^a ^*P *< 0.01, ^b ^*P *< 0.05), the MI and post-MI depression group was significantly larger than the depression group (^c ^*P *< 0.01, ^d ^*P *< 0.05), and the post-MI depression group was a significantly greater than the MI group (^e ^*P *< 0.01, ^f ^*P *< 0.05).

### The expression of Bax and Bcl-2 protein and Bax/Bcl-2 ratio

There was a significantly higher ratio of Bax:Bcl-2 (mean ± SEM) in the myocardium in the depression, MI and post-MI depression groups than in the sham group (*P *< 0.01). Furthermore, in the post-MI depression group there was a significantly higher ratio of Bax:Bcl-2 than in the depression and MI groups (*P *< 0.01), and in the MI group there was a significantly higher ratio of Bax:Bcl-2 than in the depression group (*P *< 0.01) (Table 4).

**Table 4:** The expression of Bax and Bcl-2 protein and Bax/Bcl-2 ratio (mean ± SEM, IOD)

Groups (n)	Bax	Bcl-2	Bax/Bcl-2
Sham (8)	3.32 (0.92)	2.74 (1.03)	1.14 (0.22)
Depression (8)	9.47 (2.5)^a^	6.18 (2.21)^a^	1.55 (0.30)^a^
MI (13)	27.63 (5.61)^a, b^	9.42 (3.75)^a^	2.83 (0.51)^a, b^
Post-MI depression (7)	53.88 (16.18)^a, b, c^	14.82 (3.71)^a, c^	3.77 (0.64)^a, b, c^

There was a significantly higher ratio of Bax:Bcl-2 (mean ± SEM) in the myocardium in the depression, MI (myocardial infarction) and post-MI depression groups than in the sham group (^a ^*P *< 0.01). Furthermore, in the post-MI depression and MI groups there was a significantly higher ratio of Bax:Bcl-2 than in the depression group(^b ^*P *< 0.01), and in the post-MI group there was a significantly higher ratio of Bax:Bcl-2 than in the MI group (^c ^*P *< 0.01).

### Caspase-3 levels

No difference was seen in caspase-3 levels among the sham, depression, MI and post-MI depression groups (Table 3).

## Discussion

There is an established link between depression and myocardial infarction in morbidity; however, the mechanisms underpinning this association are not comprehensively elucidated [36]. In this study, we demonstrated the behavioral phenotype of depression after myocardial infarction, characterized by lower scores of horizontal movements, vertical movements and reduced consumption of sucrose solution, in the depression and post-MI depression rats. These are models of lowered emotional activity and exploratory behavior, where the post-MI depression group demonstrated altered reward preference.

Apoptosis is an ordered, active, non-inflammatory process of cell death caused by a pathological or physiological stimulation of a genetically mediated regulatory system. Previous studies have demonstrated that in ischemia and reperfusion damage, apoptosis in the myocardium plays a role in the pathology of heart diseases, including myocardial infarction and dilated cardiomyopathy [21,37]. Cardiomyocyte apoptosis is a key form of cell death, and apoptotic cell death plays an important role in the development of heart failure [38].

There are two major apoptotic pathways in mammalian cells, ''intrinsic'' and ''extrinsic''. Both kinds of apoptotic pathways were observed simultaneously in the experiment secondary to activation and non-activation of caspase-3, which may cause cleavage of substrates and cell death. The mitochondrial-mediated pathway of apoptosis is regulated by the Bcl-2 family of antiapoptotic (Bcl-2, Bcl-xl, Mcl-1) and proapoptotic proteins (Bax, Bad and Bak), and Bcl-2 inhibits apoptosis by interacting and forming inactivating heterodimers with Bax/Bak. It has been suggested that the Bax/Bcl-2 ratio may be more important than either promoter alone in determining apoptosis. The Bax/Bcl-2 ratio is a measure of a cell's vulnerability to apoptosis; therefore, in our study, the use of a more sensitive, Bax/Bcl-2 ratio predominantly reflected apoptosis. In myocytes, the 'intrinsic'' pathway is primarily activated when cells are stimulated by hypoxia, ischemia-reperfusion and oxidative stress [26]. Oxidative stress has been hypothesized in part to mediate the link between somatic and psychiatric disorders [39]. Cardiac dysfunction and heart failure are documented after acute emotional stress [40]. The pathway of apoptosis is influenced by the Bax/Bcl-2 ratio and activated caspase-3. A high Bax/Bcl-2 ratio is associated with greater vulnerability to apoptotic activation, while a high caspase-3 level is often associated with apoptotic activity.

We observed that there was an increased myocardial Bax:Bcl-2 ratio in the depression, MI and post-MI depression groups, this was particularly so in the latter group, where there was a greater Bax:Bcl-2 ratio which is important in determining a cell's vulnerability to apoptosis. Up-regulation of the Bax/Bcl-2 ratio can induce greater apoptotic activity [16,17], suggesting that there was greater vulnerability to apoptosis of myocardial cells with acute myocardial infarction with comorbid major depression. These data suggest that post-MI depression can activate pro-apopotic pathways; however, the regulatory mechanisms underlying apoptosis in the myocardium remain unclear.

This study also demonstrated that the up-regulated Bax/Bcl-2 ratio may modulate apoptosis associated with progression of the disease [41]. The Bax/Bcl-2 ratio may serve as an independent predictive marker of the therapeutic response [42], and merits further examination, as the myocardial infarction with depression induced increase of the Bax/Bcl-2 ratio might enhance apoptosis of cardiomyocyte [43,44]. These data suggest that depression after myocardial infarction may increase the Bax/Bcl-2 ratio and induce further cardiomyocyte apoptosis, which may play an important role in the higher morbidity after myocardial infarction in conjunction with depression.

Contrary to hypotheses, we found no difference in caspase-3 in the myocardium of the post-MI depression group versus the MI, depression and sham groups. This may indicate that caspase-3 is not an active pathway to apoptosis in the myocardium in the model of myocardial infarction with depression. In interpreting this, several factors should be considered. First, caspase-3 may not be activated during the post-MI depression. It is possible that caspase-3 activity was low because the apoptotic process in the myocardium was only finished two weeks post-MI. Second, it is possible that caspase-3 activity may induce apoptosis via another independent pathway. Third, the up-regulated Bax/Bcl-2 ratio may decrease the cell's viability in involving other effector caspases without the activity of caspase-3 [16]. Other caspases may be involved. Lancel *et al*. [45] showed that endotoxin induced increases in ventricular cardiomyocyte caspase-3, -8 and -9-like activities. This was associated with sarcomeric structure damage and cleavage of components of the cardiac myofilament. Frantz *et al*. [46] noted that rats with deletion of the caspase-1 gene showed increased peri-infarct survival and a lower rate of ventricular dilatation and a decreased rate of apoptosis after a model of myocardial infarction.

However, we did not find a relationship between other caspases and the model of MI with depression. Apoptotic pathways may interact with other pathways of shared risk including ischemia and reperfusion damage, inflammatory and oxidative pathways [38,47,48] and other non-specific mechanisms, suggesting the need for further exploration of these interactions.

In this study, we found a depressive-analog anhedonia-like state in rats after myocardial infarction. These changes have parallels with the core symptoms of depression. There may be a mechanistic association with the up-regulation of the Bax/Bcl-2 ratio in the myocardium after myocardial infarction with depression. Our study demonstrated that in rats with post-MI depression, there is an increase in pro-apoptotic pathways in myocardium after myocardial infarction. Cardiomyocyte apoptosis is a key form of cell death, and apoptotic cell death plays an important role in the development of heart failure [38], resulting in a decreased heart function and reduced cardiac output.

There are certain limitations that need to be kept in mind when interpreting these data. First, our previous study demonstrated that in rats with chronic mild stress, there is an increase in pro-apoptotic pathways in the myocardium and hippocampus after depression, which was reversed by venlafaxine. To extend this line of study, our purpose was to attempt to evaluate myocardial apoptosis after myocardial infarction with depression, to clarify the molecular mechanisms, as well as confirm whether the higher incidence of myocardial infarction with depression is associated with apoptosis pathways, so the use of an inhibitor of apoptosis after myocardial infarction may further clarify the role of apoptosis. Similarly, the use of a chronic unpredictable stress-depressed (depression) group on which surgery is performed as another "control" group could have assisted interpretation of these results. Second, in our experiment, only 7 out of 20 myocardial infarction rats developed an anhedonic-like state compared to sham and depression groups; this pattern differs from reports by other groups and may be related to methodological variance. Third, it would also have been helpful for the sucrose test to have been done weekly in addition to baseline and endpoint readings. Further experimentation will clarify this point. In addition, although our results showed no effect of caspase-3 level in the myocardium after myocardial infarction and depression, it may induce apoptosis via an independent caspase-3 pathway, or other caspases expressed in the heart, suggesting again the need for further study.

## Conclusions

The results of this animal model suggest that active pro-apoptotic pathways may be involved in the nexus between cardiovascular disorders and depression. The operative mechanism might be the effects of apoptosis vulnerability markers on the myocardium in the model of depression after myocardial infarction. Apoptotic pathways may interact with other pathways of shared risk including ischemia and reperfusion damage, inflammatory and oxidative pathways, and other non-specific mechanisms. These data nevertheless suggest that active pro-apoptotic pathways may be involved in the morbidity after myocardial infarction in those with MDD. This mechanism may be germane to understanding this relationship in humans. A clear understanding of these pathways could shed light on the potential protective effects from further heart damage after myocardial infarction with depression, as well as their effects on common pathways to medical comorbidity.

## Abbreviations

APA: American Psychological Association; CAD: coronary artery disease; CMS: chronic mild stress; DEPC: diethypyrocarbonate; ECG: electrocardiogram; MDD: major depressive disorder; MI: myocardial infarction; NIH: National Institute of Health; PBS: phosphate buffered saline; RT-PCR: reverse transcription - polymerase chain reaction; SNRI: serotonin-norepinephrine reuptake inhibitor; TTC: 2,3,5-triphenyl tetrazolium chloride

## Competing interests

MB has received Grant/Research Support from the NIH, Cooperative Research Centre, Simons Autism Foundation, Cancer Council of Victoria, Stanley Medical Research Foundation, MBF, NHMRC, Beyond Blue, Geelong Medical Research Foundation, Bristol Myers Squibb, Eli Lilly, Glaxo SmithKline, Organon, Novartis, Mayne Pharma and Servier; and has been a speaker for Astra Zeneca, Bristol Myers Squibb, Eli Lilly, Glaxo SmithKline, Janssen Cilag, Lundbeck, Merck, Pfizer, Sanofi Synthelabo, Servier, Solvayand Wyeth; and served as a consultant to Astra Zeneca, Bristol Myers Squibb, Eli Lilly, Glaxo SmithKline, Janssen Cilag, Lundbeck and Servier. The other authors declare that they have no conflicts of interest.

## Authors' contributions

YW conceived of the study. XL participated in the design of the study and performed the statistical analysis. MB and YW participated in the sequence alignment and drafted the manuscript. MB critically revised the manuscript for important intellectual content. DZ, JC and SL carried out behavioral tests, molecular genetic studies and immunohistochemistry tests. All authors participated in data interpretation, drafting of the manuscript and have read and approved the final manuscript.

## Pre-publication history

The pre-publication history for this paper can be accessed here:

http://www.biomedcentral.com/1741-7015/11/32/prepub
